# The Basal Complex Protein PfMORN1 Is Not Required for Asexual Replication of Plasmodium falciparum

**DOI:** 10.1128/msphere.00895-21

**Published:** 2021-12-08

**Authors:** Colleen J. Moran, Jeffrey D. Dvorin

**Affiliations:** a Division of Infectious Diseases, Boston Children’s Hospital, Boston, Massachusetts, USA; b Department of Pediatrics, Harvard Medical School, Boston, Massachusetts, USA; University of Georgia

**Keywords:** *Plasmodium falciparum*, cell division, malaria

## Abstract

Plasmodium falciparum, the Apicomplexan parasite that causes the most severe form of human malaria, divides via schizogony during the asexual blood stage of its life cycle. In this method of cell division, multiple daughter cells are generated from a single schizont by segmentation. During segmentation, the basal complex forms at the basal end of the nascent daughter parasites and likely facilitates cell shape and cytokinesis. The requirement and function for each of the individual protein components within the basal complex remain largely unknown in P. falciparum. In this work, we demonstrate that the P. falciparum membrane occupation and recognition nexus repeat-containing protein 1 (PfMORN1) is not required for asexual replication. Following inducible knockout of PfMORN1, we find no detectable defect in asexual parasite morphology or replicative fitness.

**IMPORTANCE** Plasmodium falciparum parasites cause the most severe form of human malaria. During the clinically relevant blood stage of its life cycle, the parasites divide via schizogony. In this divergent method of cell division, the components for multiple daughter cells are generated within a common cytoplasm. At the end of schizogony, segmentation partitions the organelles into invasive daughter parasites. The basal complex is a ring-shaped molecular machine that is critical for segmentation. The requirement for individual proteins within the basal complex is incompletely understood. We demonstrate that the PfMORN1 protein is dispensable for blood stage replication of P. falciparum. This result highlights important differences between *Plasmodium* parasites and Toxoplasma gondii, where the ortholog T. gondii MORN1 (TgMORN1) is required for asexual replication.

## INTRODUCTION

Human malaria is an important and ongoing cause of global morbidity and mortality. The majority of the 200 million cases and 400,000 deaths attributable to malaria annually are due to infection by Plasmodium falciparum parasites (1). Following the infectious bite of a female *Anopheles* mosquito, P. falciparum parasites travel to the liver, differentiate, replicate, and are released as merozoites capable of invading red blood cells. During the clinically important blood stage of human malaria, P. falciparum parasites replicate asexually within red blood cells through a process known as schizogony. In this method of replication, the parasite nuclei and associated organelles undergo several rounds of division without cytokinesis (2). In the final stage of schizogony, known as segmentation, the nuclei and required organelles are partitioned with high fidelity into daughter merozoites followed immediately by cytokinesis (3). The inner membrane complex (IMC), a flattened vesicle with associated proteins that lies interior to the nascent merozoite plasma membrane (4), and the basal complex, a group of proteins that forms a ring at the leading edge of the IMC (5), are two essential structures for segmentation. The IMC, together with the subpellicular alveolin filaments, is hypothesized to provide shape and structural stability to the merozoite, aid in parasite cell division, and facilitate gliding motility during invasion (6–16). The basal complex is hypothesized to serve as the contractile ring that mediates cytokinesis (17–19).

The membrane occupation and recognition nexus protein 1 (MORN1), identified in Toxoplasma gondii, was the first component of the basal complex to be identified in Apicomplexan parasites (20). T. gondii MORN1 (TgMORN1) localizes to the basal/leading edge of the IMC and, additionally, to the centrosome and the apical end of T. gondii parasites. Conditional disruption of TgMORN1 has profound effects on parasite cytokinesis with formation of multiheaded parasites that are connected at their basal ends (18, 21). Additional basal complex proteins have been identified in both T. gondii (T. gondii DLC [TgDLC] [22], TgCentrin2 [19], TgIMC5, TgIMC8, TgIMC9, TgIMC13, TgIMC15 [23], Tg14-3-3, TgMSC1a [24], TgDIP13 [25], and TgMyoJ [26]) and P. falciparum (P. falciparum BTP1 [PfBTP1] [27], PfCINCH, PfBTP2, PfBCP1 [5], and PfHAD2 [28]). Some basal complex proteins are present in both P. falciparum and T. gondii (e.g., Tg/PfMORN1 [17] and TgHAD2a/PfHAD2 [28]). However, multiple basal complex proteins identified P. falciparum are absent in T. gondii, including PfBTP1, PfBTP2, PfBCP1, and PfCINCH, indicating divergence between the two Apicomplexan parasites. While P. falciparum MORN1 (PfMORN1) is known to be part of the basal complex in P. falciparum (5, 17, 27, 29), its functional requirement for asexual replication remains unknown. In the current study, we investigate the functional requirement of PfMORN1 (PF3D7_1031200) for asexual replication of P. falciparum.

## RESULTS

### PfMORN1 is a member of the *Plasmodium* basal complex.

**FIG 1:**
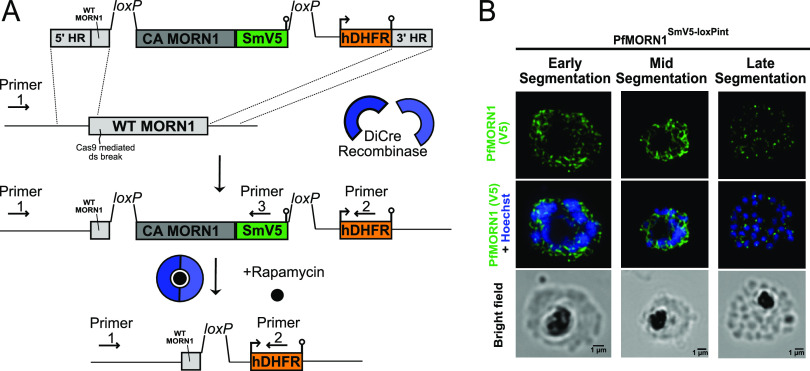
PfMORN1 is a member of the *Plasmodium* basal complex. (A) Schematic of the loxPint system for excision of the *PfMORN1* genomic locus. The location of the homology regions, Cas9-mediated double-strand (ds) break, and human dihydrofolate reductase (hDHFR) selectable marker are shown. To clearly show the location of each feature, the schematic is not drawn to scale. WT, wild type. (B) Airyscan superresolution microscopy of PfMORN1 throughout segmentation. The basal complex forms as a ring in the apical pole of the segmenting daughter cell. The complex progresses as a ring down the length of the daughter cell before converging to form punctate dots at the basal pole of the fully segmented daughter cell. Bars, 1 μm.

To study the localization of PfMORN1, we fused a spaghetti monster V5 (SmV5) (30) epitope tag to the carboxy terminus of PfMORN1 (Fig. 1A) at the endogenous locus. As expected, PfMORN1-SmV5 forms rings around nascent daughter cells in segmenting schizonts by immunofluorescence (Fig. 1B). The PfMORN1 ring is first visualized in early segmentation, enlarges during mid-segmentation, and constricts to a punctate spot at the end of segmentation—a localization pattern consistent with previously identified members of the basal complex (5, 27). Together with previous coimmunoprecipitation data using PfCINCH (5), these results confirm that PfMORN1 is a bona fide member of the P. falciparum basal complex.

### PfMORN1 is efficiently knocked out within a single asexual cycle.

To interrogate the function of PfMORN1, we utilized the loxPint inducible knockout system (31). As noted above, we introduced a recodonized PfMORN1 coding sequence fused to the SmV5 epitope tag. This cassette was flanked by *loxP* sequences nestled in synthetic introns (loxPint), with the first loxPint site introduced 99 bp into the PfMORN1 coding sequence. This strain was generated in the 3D7-*pfs47*DiCre parasite line that expresses both halves of a dimerizable Cre recombinase (32). The resulting transgenic parasite strain was named PfMORN1^SmV5-loxPint^ (Fig. 1A). The addition of the small molecule rapamycin (rapa) causes dimerization of the two halves of the Cre recombinase enzyme, allowing efficient excision of the *loxP*-flanked DNA sequences. Thus, following the addition of rapamycin to PfMORN1^SmV5-loxPint^ parasites, the activated DiCre recombinase excises >90% of the PfMORN1 coding sequence and the SmV5 epitope tag (see below for efficiency of excision), leading to a functional PfMORN1 protein knockout.

**FIG 2:**
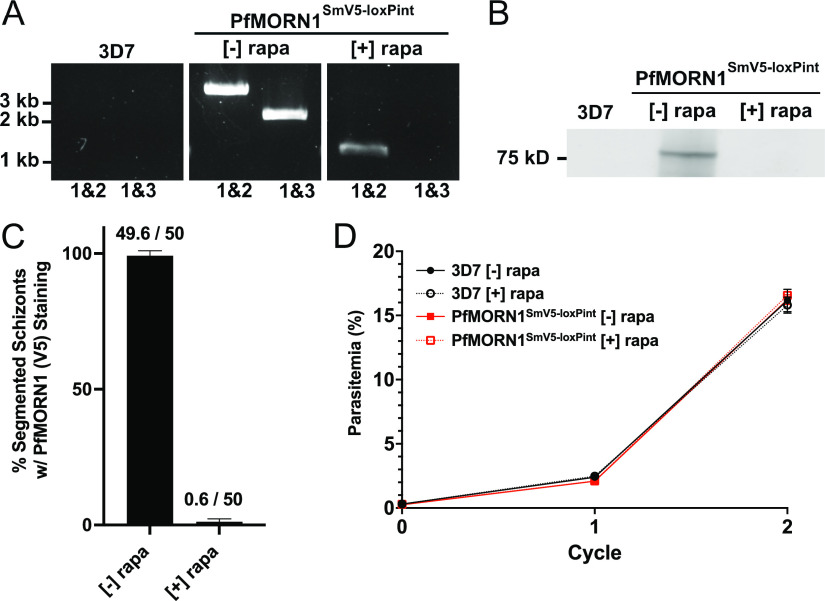
PfMORN1 is efficiently knocked out in one asexual cycle and is not required for asexual replication. (A) Agarose gel showing PCR products amplified from genomic DNA of parasites grown in the absence of rapamycin ([−] rapa) and in the presence of rapamycin ([+] rapa). (B) Immunoblot of PfMORN1 protein levels [−] and [+] rapa. The uncropped blot and total protein staining are shown in Fig. S1 in the supplemental material. (C) Quantification of the presence of V5 staining in actively segmenting (PfGAP45-positive) schizonts [−] and [+] rapa. Values are means plus standard deviations (SD) (50 schizonts counted, *n* = 3). (D) Replication curve of PfMORN1^SmV5-loxPint^ parasites [−] and [+] rapa over two replicative cycles. Means ± SD are shown (*n* = 3).

Sorbitol-synchronized ring stage parasites were treated with 100 nM rapamycin to induce excision. At the schizont stage, genomic DNA (gDNA) was evaluated by PCR to confirm excision of the *loxP*-flanked sequence from the parasite genome. The locations of the primers used for PCR are shown in Fig. 1A. We paired primer 1, which binds in the 5′ untranslated region (UTR) of PfMORN1 and upstream of the 5′ loxPint site, with primer 2, which binds downstream of the 3′ loxPint site (Fig. 1A). With this primer set, a lack of recombination results in an amplification product of approximately 3.5 kb, and successful excision results in a product of a reduced size, approximately 1 kb. We also paired primer 1 with primer 3, which binds within the flanked loxPint sites (Fig. 1A). With this primer set, a lack of recombination results in an amplification product of approximately 2 kb, and successful excision results in no PCR product. We collected gDNA from late schizonts maintained in the absence and presence of rapamicin ([−]/[+] rapa) from the ring stage of the same cycle. As a control, the wild-type 3D7 gDNA resulted in no PCR products, as expected since both reverse primers 2 and 3 sit within the genetically altered construct. In [−] rapa conditions of PfMORN1^SmV5-LoxPint^ parasites, we observe that primers 1 and 2 (primers 1&2) produce a 3.5-kb PCR product and primers 1&3 produce a 2-kb product, suggesting no excision occurred. In [+] rapa conditions, we observe primers 1&2 produce a 1-kb PCR product and primers 1&3 result in no PCR products, demonstrating that efficient excision occurs upon addition of rapamycin (Fig. 2A).

10.1128/msphere.00895-21.2 FIG S1 (A) Uncropped immunoblot from Fig. 2B. (B) A second polyacrylamide gel (4 to 20% mini-Protean TGX stain-free gels [Bio-Rad]) was run in parallel and visualized after activation by UV illumination. Download FIG S1, PDF file, 0.3 MB. Copyright © 2021 Moran and Dvorin. 2021 Moran and Dvorin. https://creativecommons.org/licenses/by/4.0/ This content is distributed under the terms of the Creative Commons Attribution 4.0 International license.

To examine efficiency of PfMORN1 protein knockout, we collected late schizonts maintained on [−]/[+] rapa from ring stage of the same cycle and performed an immunoblot probing with an antibody against the V5 epitope. Endogenous PfMORN1 is predicted to be ∼41 kDa, and SmV5 adds an additional ∼44 kDa. The expected ∼85-kDa band is present in the [−] rapa lysate and is undetectable in the [+] rapa lysate (Fig. 2B). In addition, we collected schizonts maintained [−]/[+] rapa from the ring stage of the same cycle stage for immunofluorescence assay with antibodies against V5 and the IMC-associated protein PfGAP45 (33). We identified schizonts with PfGAP45 signal, indicating they are actively segmenting, and calculated the percentage of actively segmenting schizonts with observed PfMORN1 (V5) staining. In [−] rapa conditions, we observe 99.2% ± 0.8% of actively segmenting schizonts with PfMORN1 staining. In [+] rapa conditions, we observe an average of 1.20% ± 0.49% of actively segmenting schizonts with PfMORN1 staining, a >98% reduction in PfMORN1 detection by immunofluorescence after addition of rapamycin (Fig. 2C). These results demonstrate efficient excision following rapamycin treatment.

### PfMORN1 is not required for asexual proliferation of P. falciparum.

To examine the consequence of PfMORN1 deficiency on asexual proliferation, we performed a flow cytometry-based growth assay over two complete intraerythrocytic development cycles. Rapamycin ([+] rapa) or dimethyl sulfoxide (DMSO) ([−] rapa) was added to rings during cycle 0. Parasitemia was assessed by flow cytometry, after staining with SYBR green I, upon initial seeding (cycle 0) and for the following two replicative cycles. Over two asexual cycles, the parasitemia of parasites under [+] rapa conditions were not significantly different from those under [−] rapa conditions (Fig. 2D), and both were similar to the control 3D7 wild-type strain.

**FIG 3:**
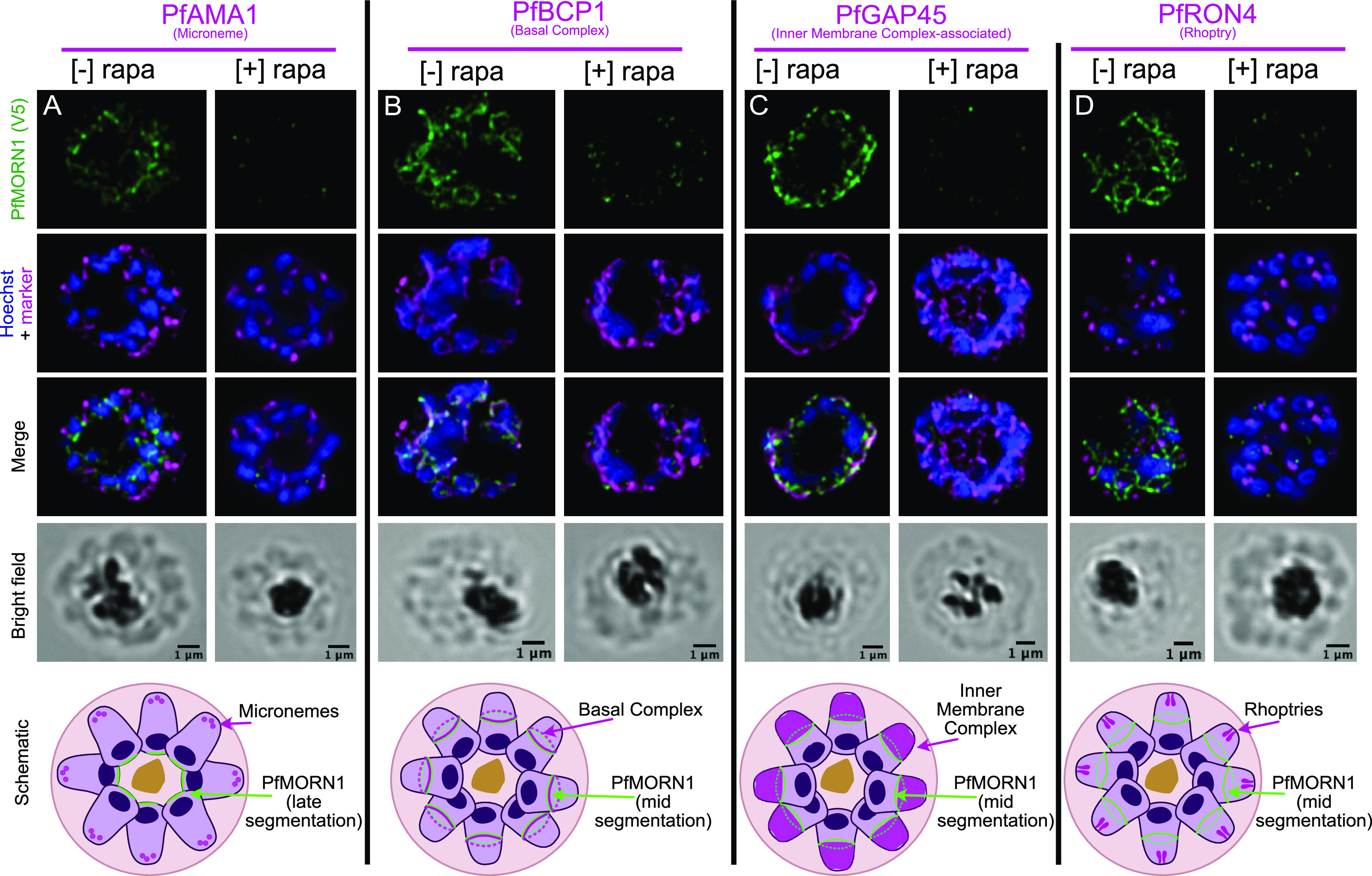
PfMORN1 excision is not required for proper morphological localization of proteins markers for several major subcellular structures. Airyscan superresolution microscopy of PfMORN1 (V5) and PfAMA1 (A), PfBCP1 (B), PfGAP45 (C), and PfRON4 (D) in [−] and [+] rapa schizonts. A schematic of the localization for each marker is shown below each column. A late segmentation schizont is shown for panel A because PfAMA1 can be confidently visualized only at this stage. For the other markers, mid-segmentation schizonts are shown. Bars, 1 μm.

To examine the impact of PfMORN1 deficiency on major subcellular structures, we collected schizonts maintained [−]/[+] rapa from the ring stage of the same cycle for immunofluorescence assay. We probed with antibodies specific for V5 and four other proteins that are markers of major subcellular structures within the parasite: PfAMA1 (micronemes) (34), PfBCP1 (basal complex) (5), PfGAP45 (IMC) (33), and PfRON4 (rhoptries) (35). In the absence of PfMORN1, PfAMA1 still exhibits apical localization that is typically observed prior to egress (Fig. 3A). Confirming that micronemal PfAMA1 staining appears very late into the segmentation process, in [−] rapa parasites, PfMORN1 (V5) staining is observed only near the basal end of segmenting parasites when PfAMA1 staining is present. Despite being a member of the basal complex, PfMORN1 deficiency has no impact on the localization of the basal complex member PfBCP1, which forms rings around segmenting parasites in the absence of PfMORN1 (Fig. 3B). Similarly, in the absence of PfMORN1, PfGAP45 still surrounds segmenting daughter parasites from the apical end down to the basal complex, observed as a ring in a single z-slice (Fig. 3C). Finally, in the absence of PfMORN1, PfRON4 still localized as punctate dots at the apical end of the forming merozoites (Fig. 3D), suggesting normal morphology of rhoptries. These results demonstrate that PfMORN1 is not required for asexual parasite proliferation and that PfMORN1-deficient schizonts (and fully formed merozoites) are morphologically normal.

## DISCUSSION

The basal complex is essential for the asexual proliferation of P. falciparum (5) and T. gondii (18, 19, 21, 26). Moreover, the basal complex is hypothesized to form a contractile ring that facilitates, or even mediates, cytokinesis of nascent daughter parasites (18, 19, 21, 26). The methods of cytokinesis during the asexual stages of P. falciparum (schizogony) and T. gondii (endodyogeny) differ, and the requirements for individual components of the basal complex likely also differ (2). However, it remains unknown which members of this multiprotein complex are redundant and/or dispensable for the P. falciparum intraerythrocytic development cycle. Dissecting which members of the basal complex are essential is an important step toward understanding the molecular functions and mechanisms of the basal complex. In T. gondii, TgMORN1 has an important role in endodyogeny, specifically for proper cytokinesis of daughter parasites (18, 21). In contrast, the current study demonstrates that PfMORN1 is dispensable for daughter cell cytokinesis during schizogony. In the P. falciparum genome-wide transposon mutagenesis screen, there were no piggyBac insertions in the PfMORN1 coding sequence (36). However, the P. berghei PlasmoGEM knockout screen predicted that PbMORN1 (PBANKA_0515200) was dispensable in the mouse model (37). The use of a rapid (i.e., single cycle) inducible knockout system likely provides strong protection against the development of compensatory mutations. Given the lack of phenotype following knockout, it is difficult to further determine the molecular function of this protein during schizogony. It is important to note that PfMORN1 may be essential for a different stage of the P. falciparum life cycle or more important for asexual replication *in vivo.* Further studies are needed to investigate these potentials roles of PfMORN1 in other environments.

## MATERIALS AND METHODS

### Reagents and antibodies.

Primers were obtained from Life Technologies, restriction enzymes from New England Biolabs, and DNA polymerases from Clontech. Commercially available antibodies were obtained from Bio-Rad (mouse anti-V5, clone SV5-Pk2) and Immunology Consultant Laboratories (rabbit anti-V5, clone RV5-45A-Z). Other primary antibodies were generously provided by Julian Rayner at the Cambridge Institute for Medical Research (rabbit anti-PfGAP45) (33), Alan Cowman, Jenny Thompson, and Kaye Wycherley at The Walter & Eliza Hall Institute of Medical Research (mouse anti-PfRON4) (35), and Robin Anders at The Walter & Eliza Hall Institute of Medical Research (mouse anti-PfAMA1 clone 1F9) (38). The primary rabbit anti-PfBCP1 antisera has been described previously (5).

### Plasmodium falciparum culturing and transfection.

The 3D7-*pfs47*DiCre parasite strain, obtained from Ellen Knuepfer at The Francis Crick Institute, was maintained *in vitro* in human O+ erythrocytes at 4% hematocrit in RPMI 1640 (Sigma) supplemented with 23 mM HEPES [4-(2-hydrocyethyl)-1-piperazineethanesulfonic acid] (EMD Biosciences), 0.21% sodium bicarbonate (Sigma), 50 mg/liter hypoxanthine (Sigma), and 0.5% Albumax II (Life Technologies).

For CRISPR transfection, 50 μg of the homology-directed repair (HDR) plasmid was linearized by digestion, purified, and cotransfected with 50 μg each of two Cas9 and guide RNA-expressing plasmids, each expressing a different guide, into sorbitol-synchronized parasites. One day posttransfection, parasites were selected with 2.5 nM WR99210 (Jacobus Pharmaceuticals). Transgenic parasites were cloned by limiting dilution, and integration of the targeting construct was confirmed by PCR with oligonucleotides oJDD5078/oJDD5079 (control), oJDD1092/oJDD4709 (DiCre integration), oJDD2933/oJDD5401 (3′ integration), and oJDD56/oJDD5402 (5′ integration). All sequences for oligonucleotides are provided in Table S1 in the supplemental material.

10.1128/msphere.00895-21.1 TABLE S1 Sequences for the oligonucleotides and synthesized gene block utilized in this study. Download Table S1, DOCX file, 0.02 MB. Copyright © 2021 Moran and Dvorin. 2021 Moran and Dvorin. https://creativecommons.org/licenses/by/4.0/ This content is distributed under the terms of the Creative Commons Attribution 4.0 International license.

### Plasmid construction.

To create the loxPint PfMORN1 HDR plasmid, we synthesized a gene block with a codon-altered PfMORN1 sequence (gBlock from Integrated DNA technology). Oligonucleotides oJDD5204/oJDD5205 and oJDD5206/oJDD5207 were used to amplify this gene block and introduce a point mutation that removed a BsaI-cut site. These fragments were fused by overlapping PCR. The PF3D7_1031200 3′ and 5′ homology regions, respectively, were PCR amplified from genomic DNA with oligonucleotides oJDD5200/oJDD5201 and oJDD5202/oJDD5203. The SmV5 epitope tag was amplified with oligonucleotides oJDD5208/oJDD5224 from pRR92 (5). The drug selection cassette (loxPint-3’UTR-Cam 5’UTR-hDHFR-hrp2UTR) was amplified with oligonucleotides oJDD5225/oJDD3907. The pGEM plasmid backbone (after site-directed mutagenesis to remove existing BsaI sites) was amplified with oJDD5227/oJDD5228. The six pieces were ligated via golden gate cloning by 150 cycles with 1 cycle consisting of 5 min of digestion with BsaI-HFv2 and 5 min of ligation with T4 ligase to generate pCJM17. All oligonucleotide and synthesized gene block sequences are shown in Table S1.

### PfMORN1 depletion.

In all PfMORN1 knockout assays, PfMORN1^SmV5-loxPint^ and parental line parasites were synchronized as rings with 5% (wt/vol) sorbitol. After one cycle, synchronized parasites were split, with half placed in 100 nM rapamycin and half with 0.02% dimethyl sulfoxide (DMSO) (32, 39).

### Western blot analysis.

Parasite protein pellets were collected from parental parasites and PfMORN1^SmV5-loxPint^ parasites in both [+] rapa and [−] rapa conditions. Proteins were extracted using 0.2% saponin in phosphate-buffered saline (PBS) with protease inhibitor, washed with PBS, and resuspended in Laemmli buffer. Samples were run on a 4 to 20% mini-Protean TGX stain-free gels (Bio-Rad). The gel was imaged to analyze total protein loading and then transferred to a nitrocellulose membrane. The membrane was blocked in Li-Cor Odyssey blocking buffer, incubated with primary antibody (1:3,000 anti-V5) in PBS with 3% bovine serum albumin (3% BSA/PBS), and then incubated in secondary antibodies diluted in Tris-buffered saline with Tween 20 (TBST). The membrane was visualized on a Li-Cor Odyssey CLx imager. Uncropped Western blot and total protein staining images are provided in Fig. S1 in the supplemental material.

### Flow cytometry analysis of parasite replication.

3D7 parental parasites and PfMORN1^SmV5-LoxPint^ parasites were synchronized as early rings using 5% (wt/vol) sorbitol and plated in triplicate at 0.25% parasitemia in 1% hematocrit in both [+] and [−] rapa conditions. One hundred microliters of each sample was collected in triplicate on days 0, 2, and 4 after plating. The cells were washed once with 0.5% BSA/PBS and then incubated for 20 min with 1:1,000 SYBR green. Cells were washed with 0.5% BSA/PBS and then resuspended in PBS. The proportion of infected cells was measured by flow cytometry using a BD FACSCalibur.

### Immunofluorescence assay.

Dried blood smears were fixed to slides in 4% paraformaldehyde for 10 min, washed three times with 1× PBS, permeabilized with 0.1% Triton X-100 in PBS, and washed again three times with 1× PBS. Slides were blocked in 3% BSA/PBS for 1 h at room temperature. Primary antibody was diluted into 3% BSA/PBS, and slides were incubated with primary at 4°C overnight. The primary antibodies and dilutions for primary antibodies were as follows: anti-V5 (1:500), anti-PfAMA1 (1:200), anti-PfBCP1 (1:250), anti-PfGAP45 (1:5000), and anti-PfRON4 (1:200). The slides were washed three times with 1× PBS and were incubated for 45 min at room temperature with secondary antibodies diluted in 3% BSA/PBS. The slides were washed three times in 1× PBS then incubated with Hoechst 33342 diluted in PBS for 10 min at room temperature. Slides were washed three times with 1× PBS and mounted using Vectashield Vibrance. Cells were visualized on Zeiss LSM880 with Airyscan with 63× oil immersion objective.
