# Supplementary material for: The Basal Complex Protein PfMORN1 Is Not Required for Asexual Replication of Plasmodium falciparum
Source: mSphere. 2021 Dec 8;6(6):e00895-21. doi: 10.1128/msphere.00895-21 (PMC8653832; doi:10.1128/msphere.00895-21)
Supplement: FIG S1 [file msphere.00895-21-sf001.pdf]

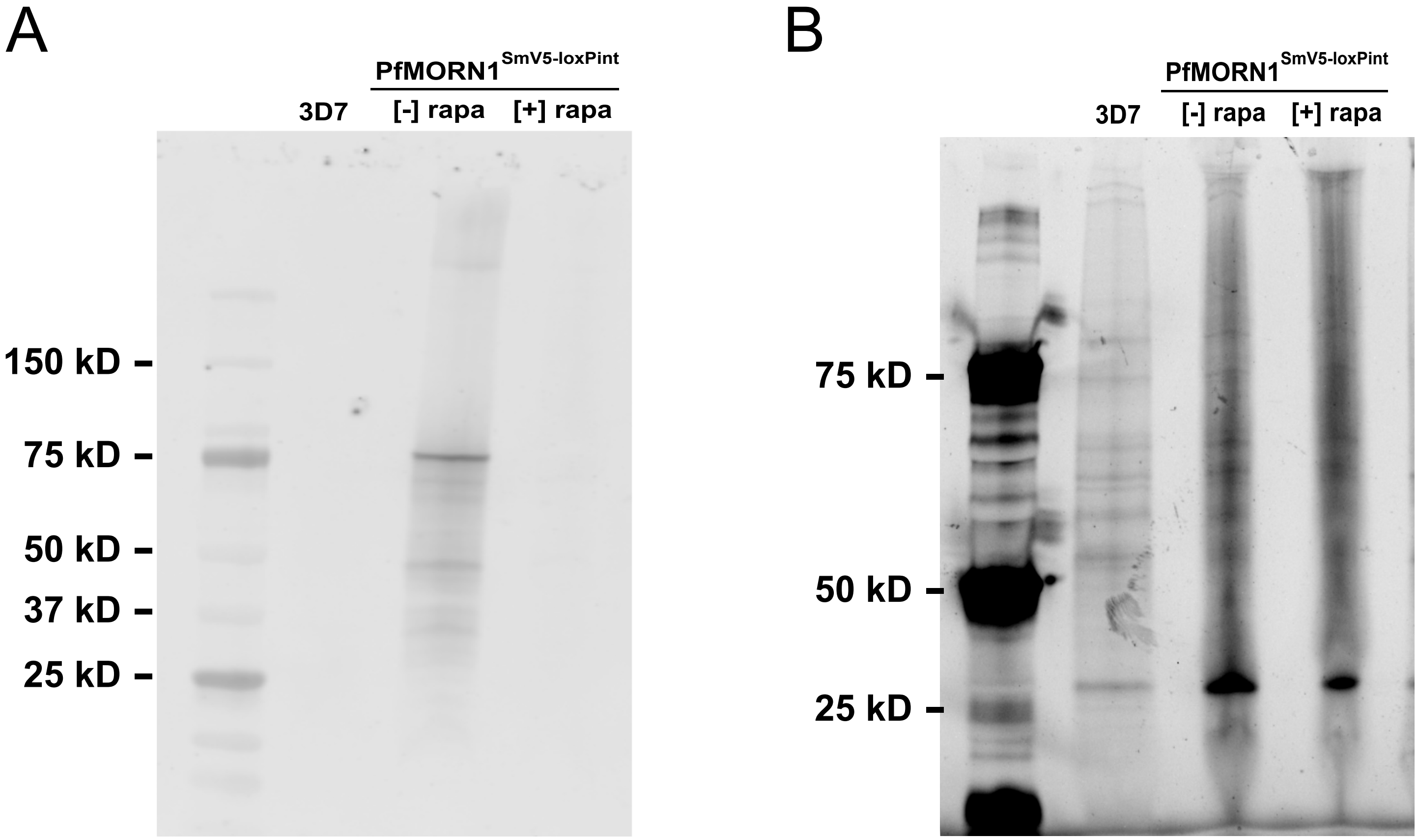

Supplementary Figure S1. **A)** This image shows the uncropped immunoblot from figure 2B. **B)** A second polyacrylamide gel (4-20% mini-Protean TGX stain-free gels (Bio-Rad)) was run in parallel and visualized after activation by UV illumination.
