# Supplementary material for: The Basal Complex Protein PfMORN1 Is Not Required for Asexual Replication of Plasmodium falciparum
Source: mSphere. 2021 Dec 8;6(6):e00895-21. doi: 10.1128/msphere.00895-21 (PMC8653832; doi:10.1128/msphere.00895-21)
Supplement: TABLE S1 [file msphere.00895-21-st001.docx]

Supplementary Table 1: Oligonucleotides and synthesized gene blocks

| **Oligo/gBlock Name** | **Sequence** |
| --- | --- |
| oJDD56 | ACACTTTATGCTTCCGGCTCGTATGTTGTG |
| oJDD1092 | AGAGTGAAGGACATCTCCCGCACCG |
| oJDD2933 (Primer 3) | CTGCTGCTGAGTACTATCAAGTC |
| oJDD3907 | GTACAGGGTCTCGAAGCGAATTCTAGATTTAATAAATATGTTCTTATATATAATGAG |
| oJDD4709 | CCACCTTGTTCACAAATACATC |
| oJDD5078 | GTACAGGGTCTCGGGAGATTCTATAAACCTGTTAGGAATAAAGG |
| oJDD5079 | GTACAGGGTCTCGAGTATTAGGATATTTTGATTCTTCAATAAACC |
| oJDD5200 | GTACAGGGTCTCGGGAGGCGGCCGCGAAGGAGAATGGTTAGATGATCATAGAC |
| oJDD5201 | GTACAGGGTCTCGTCAGGCCTCTACCGCGGCAAATTTGACAAGGTTTACGGGTAGTAC |
| oJDD5202 | GTACAGGGTCTCGCTGAAGGTGGCAATGTGTACATAGAATAACCAGTGCTACG |
| oJDD5203 | GTACAGGGTCTCGATAACTTCGTATAATGTATGCTATACGAAGTTATTGTATATTATTTTTTTTATTTACTCCTTCATATTTTTCATGCTGCG |
| oJDD5204 | GTACAGGGTCTCGTTATTATATATGTATATATATATATATTTATATATTTTATATTCTTTTAGGACTTTGTTTATGGCAAGCGCGAAGG |
| oJDD5205 | CTCGTAAGTTTCTGCGATTTTGTTCTCGTTGCC |
| oJDD5206 | GAACAAAATCGCAGAAACTTACGAGGGGGATTGGGTAGATGGTAAAATGCAAG |
| oJDD5207 | GTACAGGGTCTCGACTACCTCCCCATGGCAAATCAGGGTCGTTCCACGGCGAGG |
| oJDD5208 | GTACAGGGTCTCGTAGTATGGGAAAACCTATACCGAACCCCCTC |
| oJDD5224 | GTACAGGGTCTCGCTTCGTATAATGTATGCTATACGAAGTTATTGTATATTATTTTTTTTATTTACTTAGGTACTATCCAGTCCCAGCAA |
| oJDD5225 | GTACAGGGTCTCGGAAGTTATTATATATGTATATATATATATATTTATATATTTTATATTCTTTTAGATGGTCTTCACACTCGAAGATTTCGTTGGG |
| oJDD5227 | GTACAGGGTCTCGCTCCACGCGTCAGAGTATTCTATAGTGTCACCTAAATAGCTTGGCG |
| oJDD5228 | GTACAGGGTCTCGGCTTCTCGAGGCGCCTGATGCGGTATTTTCTCCTTACGCATCTGTGCGGTATTTC |
| oJDD5401 (Primer 1) | TGACGTATATCGACATTGTAT |
| oJDD5402 | CAGTGGAGTGTTTCAAGGTCAGGTCA |
| oJDD5407 (Primer 2) | AGACCTTCATACGGGATGATGACATG |
| GB28 (codon altered PfMORN1 gBlock) | ATGACTGAGGTTACACATTGTTACAATGGCAATATCAAAGATGGGTTGTTTCATGGCTTTGGCATCTTAATCTACTCACAGCACGAAAAGTACGAAGGAGACTTTGTTTATGGCAAGCGCGAAGGTCGTGGGAAGTTCACTTATGCCGACGGCGCTACGTACGAAGGAGAATGGGTTGATGACAAGATCCACGGCAAGGGCATTGCGAATTTCGTCTCTGGGAATATCTATGAGGGTGAATGGGAAAATGGTAAGATTAATGGCTTTGGAATGCTGTGTTATAATAATGGGGACAAGTACGAGGGCGAGTGGCTGGACGGCAAGATGCACGGTCGTGGAACGTACACGTATGAAGATGGCGATGTATATATCGGTGAATGGAAAAACGATAAGCGCCACGGAAAAGGATGCGTAAAATATAAAGGCAACGAGAACAAAATCGCAGAGACCTACGAGGGGGATTGGGTAGATGGTAAAATGCAAGGACGTGGAACATACTTCTTTGCTGATGGCGGCATCTACGAAGGGGACTGGGTGGATGGGAAAATGGAGGGAAAAGGAGTTTACAAATATCTTAATGGAAACAAATACGAGGGAGAATGGATTAATGACATGAAAAATGGCTATGGAACCCTTGCGTATGTAAATGGGGAGCTTTACGAAGGTTATTGGAAGAACGACAAAGTCCACGGTAAGGGAACATTAACGTACTCGAAGGGTGATAAGTATATTGGCGAGTGGAAATACGCCAAGAAGTGTGGCGAAGGCGAACTGATCTACGCTTCAGGTGACAAATTTAAAGGTCAATGGAAAAATGACAAGGCGAATGGTTATGGTATTTTGTTATACAACAACGGTAATAAGTACGAAGGTGAATGGCTTGATGACCATCGCCACGGGATGGGCACGTTTACCTGTAAAGAAGACGGTACAATCTACTCAGGCCATTTCCAGTTCAATCGTAAGCACGGAAAAGGGACCCTTACCTTTGTCAATGGTCACATCTTACAGGGAATCTGGAACAGTGGACTGTTAGAAAAGGTGATCAATTATGAGTTGACACCTTCCTCGCCGTGGAACGACCCTGATTTG |
